# The neutrophil-to-lymphocyte ratio as a new prognostic factor in cancers: a narrative review

**DOI:** 10.3389/fonc.2023.1228076

**Published:** 2023-10-04

**Authors:** Kian Heshmat-Ghahdarijani, Vida Sarmadi, Afshin Heidari, Alireza Falahati Marvasti, Sina Neshat, Sina Raeisi

**Affiliations:** ^1^ Cardiac Rehabilitation, Cardiovascular Research Institute, Isfahan University of Medical Sciences, Isfahan, Iran; ^2^ School of Medicine, Isfahan University of Medical Sciences, Isfahan, Iran; ^3^ School of Pharmacy, Shahid Sadoughi University of Medical Sciences, Yazd, Iran; ^4^ Department of Biostatistics and Epidemiology, University of California, San Francisco, San Francisco, CA, United States

**Keywords:** neutrophil to lymphocyte ratio, NLR, cancer prognosis, malignancy, prognostic factors

## Abstract

The increasing incidence of cancer globally has highlighted the significance of early diagnosis and improvement of treatment strategies. In the 19th century, a connection was made between inflammation and cancer, with inflammation recognized as a malignancy hallmark. The neutrophil-to-lymphocyte ratio (NLR), calculated from a complete blood count, is a simple and accessible biomarker of inflammation status. NLR has also been proven to be a prognostic factor for various medical conditions, including mortality classification in cardiac patients, infectious diseases, postoperative complications, and inflammatory states. In this narrative review, we aim to assess the prognostic potential of NLR in cancer. We will review recent studies that have evaluated the association between NLR and various malignancies. The results of this review will help to further understand the role of NLR in cancer prognosis and inform future research directions. With the increasing incidence of cancer, it is important to identify reliable and accessible prognostic markers to improve patient outcomes. The study of NLR in cancer may provide valuable insights into the development and progression of cancer and inform clinical decision-making.

## Introduction

Malignancy is a leading cause of mortality globally, continuously increasing its incidence rate (1, 2). This trend highlights the significance of early diagnosis and the need to improve treatment strategies for cancer patients (3). As far back as the 19^th^ century, the connection between inflammation and cancer was established, and inflammation was recognized as one of the six hallmark biological factors affecting the initiation and development of malignancy (4). In light of this, researchers have sought to identify appropriate inflammation biomarkers to predict the long-term outcomes of cancer patients, thereby influencing the management strategy for malignancy patients (5).

Neutrophils, constituting the largest subset of white blood cells, were originally considered pivotal in immediate immune responses, particularly in infectious diseases and wound recovery. Recent investigations, however, have illuminated their nuanced role in oncogenic processes (6–9). Neutrophils have emerged as important contributors to cancer progression through both their immunosuppressive and pro-tumoral properties (10). Emerging scientific literature substantiates that neutrophils possess a dichotomous role in oncogenesis, serving either as promoters or inhibitors of tumor expansion. This complex behavior is modulated by many variables, including but not limited to the characteristics of the intratumoral microenvironment and systemic signaling pathways (10–12). Tumor-associated neutrophils (TANs) have been empirically linked to unfavorable clinical outcomes across various cancer types (13).

Furthermore, the generation of neutrophil extracellular traps (NETs) has been identified as a critical element in the pathogenesis of malignancies (14). NETs are web-like structures containing neutrophil granule proteins, histones, and decondensed chromatin filaments (15). It has been revealed that NET deposition is involved in immunosuppression, cell proliferation, and cancer-associated thrombosis. Moreover, circulating NETs favor the intravasation of tumor cells and the establishment of micrometastasis by capturing tumor cells in the bloodstream and increasing vascular permeability (16).

Existing research posits that platelets, neutrophils, and lymphocytes are integral constituents of the tumor microenvironment, influencing various facets of tumor cell proliferation and invasion (17). Counts of these hematological components, including platelets and neutrophils alongside lymphocytes, can be readily assessed through a complete blood count (CBC).

CBC is a commonly used and readily available test in medical diagnostics and has been utilized to monitor patients. However, new utilization of CBC has been discovered, such as neutrophil-to-lymphocyte ratio (NLR) as a simple, easy-to-access biomarker of inflammation status calculated by CBC items, representing the proportion of neutrophils to lymphocytes (18). Additionally, NLR has been proven to be an effective predictive factor in several medical conditions, including the mortality classification in cardiac patients (19, 20), infectious diseases (21), postoperative complications (22), and inflammatory conditions (23). A considerable amount of literature has suggested that NLR can be considered a prognostic factor for various malignancy conditions (24–26).

This study will review recent research evaluating the relationship between NLR and various malignancies.

## Thyroid cancer

Thyroid cancer is the most common endocrine cancer, accounting for 3% of all cancer cases and nearly 96% of all endocrine cancers. The histological types of thyroid cancer include papillary, follicular, medullary, and anaplastic thyroid carcinoma (ATC), with differentiated histologic types generally having a better prognosis compared to ATC. However, evidence suggests that differentiated histologic types may also carry a risk of local and distant metastasis, which can be fatal. Thus, early recognition of patients at risk for poor prognosis is crucial.

Recently, studies have shown that the inflammatory system response plays a significant role in the initiation and promotion of thyroid cancer (27).

Inflammatory biochemical markers, such as the peripheral blood NLR, may help predict individual outcomes and evaluate treatment response. A meta-analysis of 3081 patients revealed that a high NLR is not significantly correlated with poor disease-free survival (DFS). However, subgroup analysis indicated that an increased pretreatment NLR is associated with lower DFS, especially in papillary thyroid carcinoma, where a correlation between NLR and metastasis was observed. Moreover, an elevated NLR was notably related to tumor size and metastasis (28).

Another meta-analysis of six cohorts showed that the NLR of differentiated thyroid carcinoma (DTC) patients was not significantly different from those with benign nodules. Additionally, Liu et al. found that a high NLR was not a valuable factor for predicting the development of DTC in goiter patients and that NLR did not differ between individuals younger and older than 45 years old (29).

In a study conducted in Korea, Cho et al. evaluated the ability of NLR to distinguish between poorly differentiated thyroid cancer (PDTC) and ATC, finding that a high NLR was associated with advanced stages, cancer-related mortality, and a higher proportion of PDTC to ATC (30).

In another study by Park et al., elevated NLR before and after radiotherapy (RT) was found to be significantly correlated with poor overall survival (OS) in ATC patients undergoing RT. In their study, they evaluated the predictive value of NLR before and after RT for ATC. The result revealed an elevated NLR before and after RT is considerably correlated to poor OS in ATC patients who underwent RT (31).

Lee and colleagues studied the relationship between longitudinal changes in NLR and therapy response in DTC, finding that incomplete response to therapy was associated with gender, mass size, metastasis, and increased NLR. Interestingly, they also observed a significant reduction in NLR in patients who responded well to treatment (27).

## Breast cancer

Breast cancer (BC) is a major contributor to cancer-related deaths among women globally (32). The host’s systemic inflammatory response has been shown to play a crucial role in the initiation and progression of malignancy (33). NLR is widely considered a biomarker and has been extensively studied for its potential as a predictor of outcomes in BC patients (32).

The use of neoadjuvant chemotherapy (NACT) in patients with operable primary tumors has been approved as a treatment option for BC. In a systematic review conducted by Zhou et al., 19 pieces of literature were analyzed, revealing that a low NLR was associated with higher pathological complete response (pCR) rates, while elevated NLR was linked to lower DFS and OS rates in BC patients receiving NACT (34).

In China, the correlation between NLR and the risk of BC as well as its association with obesity and metabolic syndrome (MetS) was evaluated. Patients were divided into four groups based on NLR quartiles: very low, low, medium, and elevated. Results showed that medium or elevated NLR levels were significantly correlated with BC, while the very low group did not exhibit the same correlation. The study found that a higher preoperative NLR could be associated with an increased risk of BC, regardless of menopausal status. Furthermore, an NLR value greater than 1.67 was identified as a notable risk factor for BC. Results also showed that patients without obesity or MetS were more likely to have elevated NLR levels as a risk factor for BC (35).

Ding et al. analyzed the relationship between clinicopathological variables, NLR, and DFS in human epidermal growth factor receptor 2 (HER2)-positive BC patients receiving trastuzumab therapy. Patients were divided into three categories. Pretreatment NLR was not found to have any predictive significance in Group 1, which comprised of 255 participants who did not receive trastuzumab therapy. Based on pretreatment NLR, patients receiving trastuzumab therapy were assigned to two groups: low NLR (Group 2) and elevated NLR (Group 3). Results showed that 3-year DFS was substantially higher in Group 2 compared to Group 1 and Group 3. These findings suggest that HER2-positive BC patients with lower pretreatment NLR values may respond better to trastuzumab therapy, while those with elevated NLR values may not respond as well or may respond weakly (36).

In a retrospective study conducted by Chen et al., 215 BC patients receiving NACT were analyzed. Results showed a significant difference in pCR rates between patients with low pretreatment NLR and those with elevated NLR (NLR ≥ 2.06). Patients with lower pretreatment NLR had higher pCR rates, while those with elevated NLR had higher disease-specific mortality rates and more advanced stages of cancer. Additionally, patients with elevated pretreatment NLR had substantially lower relapse-free survival (RFS) and breast cancer-specific survival (CSS) rates compared to patients with low NLR (37).

In another meta-analysis, Guo et al. investigated the predictive value of platelet-to-lymphocyte ratio (PLR) and NLR in BC individuals. Twenty-seven studies with a total of 17079 patients looked into the effect of NLR in predicting DFS. They discovered that high NLR was linked to poor DFS (32).

In a study conducted in Korea, they retrospectively analyzed the data of non-metastatic, HER2-negative BC patients who were undertreated neoadjuvant chemotherapy after receiving surgery. NLR value ≥ 2.74 was recognized as a high NLR. They demonstrated high NLR could be an indicator for DFS and OS (38).

## Lung cancer

Lung cancer (LC) is a prominent reason for malignancy mortality and accounts for 18.4% of overall cancer deaths worldwide (39). Classically, non-small cell lung cancer (NSCLC) and small cell lung cancer (SCLC) are the two main subtypes of LC (40).

Patients diagnosed with LC are often in the advanced stages of the disease, and the primary treatments include chemotherapy and surgery. Despite advances in these treatments, the response to these interventions remains unsatisfactory (41, 42). The systemic inflammatory response of the host has a significant impact on cancer development and progression, and while the inflammatory status can provide insight into LC progression, inflammatory biomarkers are not always available prior to surgery. The NLR has been shown to be a readily accessible and simple parameter for indicating systemic inflammation status (43, 44).

A study in Japan involving 111 NSCLC patients in stage 3 who received definitive chemoradiotherapy found that the average NLR and post-radiotherapy NLR were significant predictive factors for OS and DFS (45). In a meta-analysis of 16 articles, Winther-Larsen et al. found that a high NLR may be a predictive biomarker for adverse OS in SCLC patients (46).

Another meta-analysis of 2020 LC patients receiving immunotherapy found that a high NLR was strongly associated with adverse OS and progression-free survival (PFS). The subgroup analysis indicated that the post-treatment NLR was not significantly correlated with OS (47).

Huang et al. investigated the impact of the combination of NLR and preoperative fibrinogen on predicting OS in individuals with NSCLC. This study enrolled 589 NSCLC patients who underwent surgery, and the results showed that the fibrinogen-neutrophil-to-lymphocyte ratio (F-NLR) could be an independent predictive parameter for DFS and OS. The researchers observed a strong relationship between the F-NLR and tumor stage, size, and lymph node metastasis (48).

In 2017, Lan and colleagues conducted a cohort study to evaluate the prognostic potential of NLR and PLR in NSCLC patients who planned to undergo radical resection. The study enrolled 174 NSCLC patients, and the results showed that elevated PLR and NLR were significantly associated with increased postoperative pulmonary complications. Additionally, elevated NLR was strongly correlated with a high frequency of pulmonary complications (49).

## Esophageal cancer

Esophageal carcinoma is a prevalent form of cancer worldwide, with approximately 604,100 new cases recorded each year. The rapid progression and late diagnosis of esophageal carcinoma have led to high mortality and morbidity rates among patients, making early and appropriate treatment strategies a necessary consideration (50, 51). The correlation between poor prognosis and increased inflammatory response in esophageal cancer has been established, with inflammation playing a significant role in tumor initiation and development (52, 53).

In recent years, there has been a significant increase in studies exploring the use of the NLR as a predictive biomarker for the prognosis of esophageal cancer patients (54–56). Sakin et al. conducted a study in Turkey evaluating the prognostic value of NLR in 80 resectable esophageal squamous cell carcinoma patients and found that increased pretreatment NLR was correlated with decreased OS and DFS (57).

Li et al. conducted a systematic meta-analysis of 8431 patients in 32 studies and found that high NLR was significantly correlated with decreased OS, CSS, PFS, and DFS (58).

Additionally, there have been a few studies investigating the predictive role of NLR in the recurrence of esophageal cancer (59). In a retrospective cohort study conducted in Japan, the predictive value of NLR in the long-term outcome of recurrent esophageal cancer was evaluated, and the findings showed that NLR was significantly related to survival. The optimal cut-off NLR for survival was defined as 3.374 using survival classification and regression tree analysis (60).

Furthermore, recent research has revealed that not only peripheral blood NLR but also pathological tissue NLR can be a helpful predictive factor for prognosis in esophageal carcinoma patients. In a retrospective study of 103 esophageal cancer patients who underwent radical resection surgery in China, both peripheral blood and pathological tissue NLR were found to independently predict poor postoperative prognosis (61).

## Gastric cancer

Gastric cancer is a prevalent tumor that is ranked third among all malignancies worldwide in terms of death rate (39). The role of inflammation in developing gastric carcinoma has led to the evaluation of NLR as a prognostic factor in individuals with gastric malignancy. In a study by Namikawa and colleagues, the predictive usefulness of NLR and C-reactive protein-to-albumin ratio (CAR) was explored in 411 individuals with unresectable advanced or recurrent gastric cancer. The results showed a substantial correlation between high NLR and CAR with poor outcomes (62).

Another research by Liu et al. retrospectively evaluated 111 advanced gastric cancer patients undergoing NACT following curative resection. They found that a decreased level of post-NACT NLR was strongly associated with longer OS and DFS (63). In a study in Japan, the association between NLR and PLR was investigated with histological types of gastric carcinoma in patients who underwent endoscopic submucosal dissection (ESD). The results showed that patients with adenocarcinoma tend to have higher NLR and PLR levels and that undifferentiated gastric adenocarcinoma patients have higher levels of NLR and PLR compared to patients with differentiated gastric adenocarcinoma. These findings may help in predicting the indications of ESD and other treatment strategies after ESD in early-diagnosed gastric cancer patients (64).

In a study by Fu and colleagues, the prognostic role of the red blood cell distribution width-NLR (R-NLR) score was investigated in 151 stage II-III gastric cancer patients after radical resection surgery. The results showed that the R-NLR score is a favorable parameter in predicting OS in these individuals (65).

## Pancreatic cancer

Pancreatic adenocarcinoma (PAC) is a highly aggressive malignant tumor that ranks as the third and fourth leading cause of mortality among all malignancies in the United States and globally, respectively (39, 66). Despite various therapeutic measures such as surgery, neoadjuvant chemoradiation therapy, and chemotherapy, the overall long-term outcome for PAC patients remains unsatisfactory, with five-year survival rates for patients undergoing radical pancreatectomy reported as less than 27% (67). The overall prognosis of patients with PAC remains unsatisfactory, despite the implementation of various therapeutic interventions such as surgical resection, neoadjuvant chemoradiation therapy, and chemotherapy (68, 69).

In recent years, there has been a growing interest in the role of the inflammatory status and its response in tumorigenesis and the tumor microenvironment and their effect on the behavior of PAC (70–73).

Studies have been conducted to assess the prognostic significance of the pretreatment NLR and its effect on the post-treatment outcomes of PAC patients. Results of these studies have generally shown the predictive potential of NLR in various stages of the disease. However, there is still some controversy in the literature regarding the use of NLR as a prognostic marker.

In a recent cohort study conducted by Reddy et al., the predictive value of NLR before and after surgery was assessed in 156 patients with borderline resectable and locally advanced PAC. The results showed that an NLR value of more than 2.6 was significantly associated with unfavorable OS and PFS (74).

A study conducted on 226 individuals with PAC in the United States retrospectively evaluated the predictive role of NLR on survival outcomes. The results of the study indicated that high NLR was a significant independent predictor of poor prognosis in individuals with PAC. Additionally, the study showed that lower NLR values (less than 5) were more commonly seen in non-Hispanic Black patients, while non-Hispanic White and Hispanic patients tend to have higher NLR values (more than 5). These findings suggest that non-Hispanic White and Hispanic PAC patients may be at a higher risk for unfavorable outcomes (75).

However, not all studies have shown consistent results regarding the predictive potential of NLR in PAC. A retrospective cohort study conducted in 2021 showed that NLR levels increased after neoadjuvant therapy in PAC patients, but no significant correlation was observed between NLR and OS, DFS, or pathological response following treatment (76).

On the other hand, previous studies have shown that using NLR in conjunction with other biomarkers, such as CA19-9, could enhance the ability to predict outcomes in PAC (77).

In a retrospective analysis of 271 patients with end-stage PAC, Shin et al. found that a post-treatment NLR of less than 2.62 in combination with an 18% decline in CA19-9 levels was a useful predictor of outcomes. The results of the study suggest that the combination of these biomarkers may lead to a more accurate and effective prediction of outcomes in PAC patients (78).

## Hepatocellular cancer

Hepatocellular carcinoma (HCC) is a prevalent cancer, ranking seventh in frequency and third in death rate among all malignancies, with an estimated 841,080 cases and 781,631 deaths per year (79).

The Barcelona clinic liver cancer (BCLC) system is widely used for the staging and treatment of HCC and comprises five main parameters: tumor size, number, Child-Pugh class, physical state, and metastasis (80). In the early stages of HCC, curative treatments such as hepatic resection, transplantation, and radiofrequency ablation are possible, according to the BCLC system (81). However, despite advances in early HCC diagnosis, less than 30% of patients are eligible for curative treatments and the majority of patients are diagnosed at intermediate or advanced stages, with 40% diagnosed in the advanced stage (82). Currently, there is no effective treatment for severe HCC (79).

The systemic inflammatory state of an individual has a significant impact on cancer progression and tumor growth through the upregulation of cytokines. As in numerous other cancers, the NLR has been considered a marker of systemic inflammation and its predictive role in HCC patients has been explored (81).

In a meta-analysis of 20475 patients with HCC, Qi et al. found that individuals with low baseline and post-treatment NLR had significantly higher OS and RFC or DFS compared to those with high baseline and post-treatment NLR. The authors also found that individuals with a decrease in NLR had better OS than those with an increase in NLR (80).

Wu and colleagues conducted a retrospective study to evaluate the predictive value of preoperative and postoperative NLR in HCC patients who underwent partial hepatectomy. The results showed that the combination of preoperative and postoperative NLR was a valuable predictive indicator, as demonstrated by the time-dependent receiver operating characteristic curve analysis. The results also indicated that an increase in the preoperative and postoperative NLR score was associated with poor differentiation and multiple tumors (83).

Dai and colleagues conducted a study of patients with HCC who underwent liver resection to evaluate the predictive value of the change in postoperative NLR/preoperative NLR (NLRc) at different time points. The results showed that NLRc (3-6 months) could be a predictive indicator for inferior DFS among HCC patients, particularly in cirrhotic individuals. The results also indicated that NLRc (3-6 months) was significantly correlated with early recurrence, although NLR (4-8 weeks) was significantly correlated with both early and late recurrence (81).

## Colorectal cancer

Colorectal cancer (CRC) is a leading cause of cancer-related deaths worldwide and continues to pose a major public health challenge. Despite advances in early screening measures, many patients are still diagnosed in advanced stages, and the five-year survival rate among affected individuals ranges from 60 to 70% on average (84). Given the significant burden of CRC, with nearly a million deaths annually worldwide (85), it is critical to identify practical prognostic factors that can aid in the management of this disease.

Inflammation has been increasingly recognized as a key factor in tumorigenesis and neoplastic processes. As a result, the prognostic utility of biomarkers has become a subject of growing interest (4, 86, 87).

The NLR has emerged as a promising, simple, inexpensive, and readily available serum inflammatory marker for predicting the prognosis of CRC patients. Studies have investigated the prognostic role of NLR, both alone and in combination with other prognostic markers, in patients with early and advanced CRC (88–90).

In a retrospective study of 219 patients with non-metastatic CRC conducted in Turkey in 2020, the predictive value of preoperative NLR was evaluated in relation to DFS and OS after surgical resection. Multivariate analysis revealed that a preoperative NLR greater than 2.8 was associated with poorer OS outcomes in CRC patients, while DFS was not predictive of preoperative NLR (91).

In 2021, Yang and colleagues conducted a study on non-metastatic CRC patients to examine the prognostic significance of the combination of NLR and interleukin-6 (IL-6) using receiver operating characteristic curve analysis. The results indicated that the combination of these biomarkers was a stronger predictor of DFS and OS compared to either biomarker alone. Additionally, the study demonstrated a correlation between high NLR and IL-6 levels and tumor differentiation and staging (92).

Due to the challenges of providing adequate therapeutic measures and palliative care for metastatic CRC patients, the prediction of prognosis in this population is of utmost importance. In a retrospective study, Nemoto and colleagues aimed to determine the predictive role of NLR in metastatic or unresectable advanced CRC patients in Japan. The results suggested that changes in NLR values from pre-chemotherapy to three months post-chemotherapy could be a useful predictor of OS in CRC (93).

The literature also highlights the importance of persistently high NLR values as a predictor of poor prognosis in CRC patients. A recent study in China evaluated the predictive potential of high NLR values in CRC patient outcomes. After determining optimal cut-off points for pre- and postoperative NLR values, the patients were divided into four groups based on their pre- and postoperative NLR levels (low-low, low-high, high-low, and high-high). The results showed that the high-high group had the worst PFS, followed by the high-low, low-high, and low-low groups, respectively (94).

## Renal cell carcinoma

The incidence of renal cell carcinoma (RCC) has been increasing globally and it is the seventh and ninth most common cancer among men and women, respectively (95, 96).

Despite advancements in the management and surgical strategies for RCC patients, a considerable number of patients still face recurrence, distal metastasis, and inadequate drug response, highlighting the need for reliable indicators for prognosis and metastasis in order to make informed therapy decisions (96).

The role of the inflammatory system in cancer progression has gained recognition in several malignancies, and the NLR has been established as an affordable and accessible indicator for the inflammatory system (97).

A meta-analysis study by Shao and colleagues in 2020, examining the prognostic utility of NLR in RCC, found that a high pretreatment NLR was significantly associated with unfavorable OS, DFS/PFS, and CSS in the general population. Additionally, they investigated the prognostic value of NLR in patients with metastasis who received treatment with immune checkpoint inhibitors (ICIs) and concluded that elevated levels of NLR were significantly related to inferior OS and PFS (95).

Byun et al. conducted a cohort study in Korea to examine the predictive role of preoperative NLR in RCC patients without metastasis and found that NLR could be a reliable predictor for both RFS and CSS through multivariate analysis (97).

Another study by Zhao et al. on 384 small renal cell carcinoma patients who underwent curative surgery assessed the prognostic value of preoperative NLR and demonstrated that an elevated NLR was an independent indicator for OS (98).

## Prostate cancer

The incidence of prostate cancer and its associated mortality rates have risen to become a global concern, with it being identified as the most prevalent cancer among men (99). The relationship between cancer and inflammation has been widely acknowledged and has played a significant role in the development of prostate cancer (100–102). As a result, the prognostic potential of the NLR has been evaluated as a marker of inflammation in different stages of prostate cancer.

Cao et al. conducted a retrospective cohort study of 994 localized prostate cancer patients treated with radical prostatectomy in China and found that elevated NLR was significantly correlated with better biochemical recurrence survival, but it was not a reflection of prostate cancer characteristics or the local immune microenvironment (103).

On the other hand, a study by Italian researchers showed that NLR was a significant predictor of adverse pathology and biochemical recurrence in 1258 prostate cancer patients who had undergone radical resection surgery (104).

Recently, the role of NLR in predicting outcomes in metastatic castration-resistant prostate cancer (mCRPC) patients treated with abiraterone and enzalutamide has gained attention. Guan et al. conducted a meta-analysis of 3144 mCRPC individuals from 15 cohort studies, which revealed that high NLR was remarkably correlated with poor OS in mCRPC individuals treated with abiraterone and enzalutamide. However, no significant correlation was found for PFS (105). This suggests that NLR has a stronger predictive significance for prognosis in more advanced prostate cancer compared to early-stage prostate cancer.

Furthermore, the potential of NLR as a predictor of prostate cancer diagnosis has been evaluated in patients with significant clinical characteristics of prostate cancer and prostate-specific antigen (PSA) levels less than 10 ng/ml. A study by Masuda et al. on 633 individuals with PSA levels between 4.0 and 10.0 ng/ml who underwent prostate biopsy showed that NLR was an indicator of prognosis prior to prostate needle biopsy (106). Similarly, Sun et al. revealed that the combination of NLR with prostate imaging-reporting and data system version 2 can be a predictor of prostate cancer diagnosis prior to biopsy (107). These findings might help clinicians to avoid unnecessary biopsies.

## Ovarian cancer

Ovarian cancer is a prevalent and lethal gynecological cancer with a 5-year OS rate of nearly 45.6%. Unfortunately, the majority of patients are diagnosed at an advanced stage due to the lack of effective screening methods, resulting in poor long-term outcomes. In recent years, the role of inflammation in tumor progression and development has been widely recognized in the literature. The NLR, as a readily accessible biochemical inflammatory marker, has garnered significant attention in this regard (108, 109).

In a cohort study of 72 ovarian cancer patients receiving chemotherapy, it was found that elevated NLR was associated with adverse OS and poor treatment response. High NLR was identified as a weak predictive factor in this population through multivariate analysis (110).

A retrospective study on 875 high-grade serous ovarian cancer (HGSC) patients by Feng et al. found that a high preoperative NLR level was correlated with the advanced international federation of gynecology and obstetrics stage, higher CA125 level, extensive ascites, poor cytoreduction outcomes, and chemotherapy resistance. Univariate analysis revealed a negative correlation between high NLR and OS and PFS. In the multivariate analysis, elevated NLR remained an independent prognostic factor for PFS but not OS. The results suggest that the NLR may serve as an indicator of tumor burden and clinical prognosis in HGSC patients and should be considered a prognostic indicator (111).

In another study, high red cell distribution width (RDW) and PLR were found to be negatively correlated with OS and RFS in epithelial ovarian cancer (EOC) patients. The results also showed that high preoperative RDW and NLR predicted poor OS and high RDW+NLR could be a useful parameter for stratifying HGSOC patients. Moreover, high NLR was associated with adverse RFS, stage, preoperative CA125 level, and ascites (112).

A meta-analysis that analyzed ten studies involving 2919 patients found that individuals with ovarian cancer with higher NLR had a significantly inferior OS and PFS compared to the control group (113).

## Limitations and future perspectives

A major limitation in current research on NLR in cancer prognosis is the absence of standardized methods across studies. Cut-off values, sampling timings, and methodological frameworks vary, hindering the synthesis of results into clinical practice. Furthermore, the biological complexity of the interplay between neutrophils and lymphocytes in the tumoral microenvironment is not entirely elucidated, necessitating more nuanced, mechanistic investigations. An additional limitation is that most studies are based on a single time-point assessment of NLR, which doesn’t capture the temporal fluctuations that might be critical in understanding its prognostic relevance. Finally, the clinical relevance of NLR as a stand-alone biomarker versus its utility as part of a multi-marker panel has yet to be robustly evaluated.

To redress these limitations, there is an imperative for future research to focus on standardization and validation studies, which would provide more robust cut-off values and measurement techniques to render NLR a more universally applicable prognostic marker. Longitudinal studies can offer insights into the dynamic role of NLR over time, while comparative effectiveness research could help elucidate how NLR performs in contrast to other prognostic markers. Furthermore, the potential for aggregating data through meta-analyses for each type of cancer and even conducting umbrella reviews to evaluate the overarching evidence could provide clearer guidelines for the clinical application of NLR. Such consolidated findings would be instrumental in elevating the reliability and validity of NLR, thus informing better clinical decision-making in oncology.

## Conclusion

In conclusion, the prognostic potential of the NLR in cancer has been the subject of numerous studies in recent years. The results of these studies have indicated that NLR has the potential to serve as a predictive prognostic indicator for various cancers at different stages. In particular, several studies have shown that elevated NLR levels are associated with poorer OS and PFS in patients with ovarian cancer, high-grade serous ovarian cancer, and epithelial ovarian cancer. Moreover, some studies have suggested that NLR may also be an independent predictor of PFS in multivariate analyses, while others have demonstrated its ability to assess post-treatment response. The studies that we discussed in each section have been summarized in **Table 1**.

**Table 1:** Predictive Role of Neutrophil-to-Lymphocyte Ratio (NLR) in Cancer.

Cancer Type	Study	No of Patients	Results
**Thyroid**	Feng J et al.* (28)	3081	Subgroup analysis results indicated increase in pretreatment NLR is remarkably related to lower DFS. Overall, an elevated NLR was notably related to tumor size and metastasis.
	Liu J-f et al.* (29)	7349	NLR of DTC patients was not significantly different from those with benign nodules. high NLR couldn’t be a valuable factor for developing DTC in goiter patients.
	Cho et al. (30)	3870	They observed advanced stages, cancer-related mortality, and the proportion of PDTC to ATC was notably more prevalent in the high NLR patients.
	Park J et al. (31)	40	An elevated NLR before and after RT is considerably correlated to poor OS in ATC patients who underwent RT
	Lee F et al. (27)	151	Incomplete response to therapy was correlated to gender, the largeness of mass, metastasis, and increased NLR in multivariate analysis.
**Breast**	Zhou et al.* (34)	5910	Low NLR was linked to higher pCR. An elevated NLR was correlated to lower DFS and OS in BC patients who received NACT.
	Fang Q et al. (35)	1540	The results indicated, in spite of menopausal status, in the whole population, a higher preoperative NLR could be associated with increases in BC.
	Ding N et al. (36)	843	HER2-positive BC individuals with advanced pretreatment NLR values may not respond to trastuzumab treatment or may respond weakly.
	Chen Y et al. (37)	215	Significant difference in pCR rate in individuals with low pretreatment NLR and those with advanced NLR in BC patients who underwent NACT
	Guo W et al.* (32)	17079	They discovered that high NLR was linked to poor DFS.
	Bae SJ et al. (38)	1097	NLR value ≥2.74 was recognized as a high NLR. They demonstrated high NLR could be an indicator for DFS and OS among HER2-negative patients who undertreated NACT.
**Lung**	Kanzaki H et al. (45)	111	Average-NLR and post-radiotherapy NLR were notable predictive factors for the OS and DFS among NSCLC patients in stage 3 who received definitive chemoradiotherapy.
	Winther-Larsen A et al.* (46)	7762	High NLR might be a predictive biomarker for adverse OS in SCLC patients.
	Jin J et al. (47)	2068	High NLR substantially related to adverse OS and PFS.
	Huang W et al. (48)	589	F-NLR could be an independent predictive parameter for DFS and OS.
	Lan H et al. (49)	174	Elevated PLR and NLR were significantly correlated to increased postoperative pulmonary complications.
**Esophageal**	Sakin A et al. (57)	80	Increased pretreatment NLR correlated to decreased OS and DFS.
	Li B et al.* (58)	8431	High NLR is significantly correlated to decreased OS, CSS, PFS and, DFS.
	Hoshino S et al. (60)	586	NLR is significantly related to survival in patients.
	Guo Q et al. (61)	103	Peripheral blood and pathological tissue NLR independently predict poor postoperative prognosis of the patients.
**Gastric**	Namikawa T et al. (62)	411	High NLR and CAR substantially correlate to poor outcomes.
	Liu Z et al. (63)	111	Decreased level of post-NACT is strongly associated with longer OS and DFS.
	Yasui S et al. (64)	218	Undifferentiated gastric adenocarcinoma patients have higher levels of NLR and PLR compared to patients with differentiated gastric adenocarcinoma.
	Fu L et al. (65)	151	R-NLR score is a favorable parameter in predicting OS in stage II-III gastric cancer patients.
**Pancreatic**	Reddy AV et al. (74)	156	NLR level more than 2.6 is substantially associated with unfavorable OS and PFS.
	Shusterman M et al. (75)	226	High NLR is an independent indicator of poor prognosis in individuals with PAC.
	Strong JS et al. (76)	93	No significant correlation was obtained between NLR and OS, DFS, and pathological response following the treatment.
	Cetin S et al. (77)	118	Using NLR and other biomarkers such as CA19-9 could be more powerful for predicting PAC outcomes.
	Shin K et al. (78)	271	Post-treatment NLR of less than 2.62 and 18% decline of CA19-9 combined is a useful predictive indicator.
**Hepatocellular**	Qi X et al.* (80)	20475	With a lower NLR had a considerably better OS than those with a higher NLR.
	Wu M et al. (83)	70	Preoperative NLR plus postoperative NLR was superior than other parameters as a predictive factor. They showed increasing in preoperative NLR plus postoperative NLR score could be correlated to poor differentiation and multiple tumors.
	Dai T et al. (81)	195	The results showed the NLRc (3–6 m) might be a predictive indicator for inferior DFS among HCC individuals.
**Colorectal**	Gulben K et al. (91)	219	Multivariate analysis revealed that a preoperative NLR greater than 2.8 could indicate OS in CRC. However, DFS was not predicted favorably by preoperative NLR.
	Yang Z et al. (92)	88	NLR and IL-6, when used together, are a more powerful predictor of DFS and OS in patients than NLR or IL-6 alone.
	Nemoto T et al. (93)	71	Change of NLR value in the pre-chemotherapy to 3 months post-chemotherapy period could be a good indicator in predicting OS in CRC.
	Cui M et al. (94)	146	They demonstrated the high-high group had the worst PFS, followed by the high-low, low-high and low-low, respectively.
**Renal**	Shao Y et al.* (95)	6461	In general population, high pretreatment NLR was substantially correlated with unfavorable OS, DFS/PFS, and CSS. in patients with metastasis who received treatment with immune checkpoint inhibitors (ICIs), the elevated level of NLR was notably related to inferior OS and PFS.
	Byun S-S et al. (97)	1284	Multivariate analysis suggested NLR could be a reliable predictor for both RFS and CSS.
	Zhao H et al. (98)	384	Elevated NLR is an independent indicator for OS.
**Prostate**	Cao Z et al. (103)	994	Elevated NLR significantly correlated with better biochemical recurrence survival.
	Bravi CA et al. (104)	1258	NLR significantly predicts adverse pathology and biochemical recurrence.
	Guan Y et al.* (105)	3144	The pooled data revealed that high NLR was remarkably correlated with poor OS in mCRPC individuals treated with abiraterone and enzalutamide, but no significant correlation was found for PFS.
	Masuda H et al. (106)	633	Their findings showed that NLR is an indicator of prognosis among these patients prior to prostate needle biopsy.
	Sun J et al. (107)	335	NLR, in combination with prostate imaging-reporting and data system version 2, can be a predictor of diagnosis of prostate cancer prior to biopsy.
**Ovarian**	Henriksen JR et al. (110)	72	High NLR is a weak predictive indicator in these individuals. Also, high NLR was considerably related to poor treatment response.
	Feng Z et al. (111)	875	High preoperative NLR level is related to an advanced international federation of gynecology and obstetrics stage, higher CA125 level, extensive ascites, inferior cytoreduction outcome, and resistance to chemotherapy.
	Li Z et al. (112)	654	High preoperative RDW and NLR predict poor OS in EOC (epithelial ovarian cancer) patients. Also, they suggested high RDW+NLR could be a parameter for stratifying HGSOC patients. Furthermore, the results revealed high NLR is correlated to adverse RFS, stage, preoperative CA125 level, and ascites.
	Zhu Y et al.* (113)	2919	They discovered that individuals with ovarian cancer who had a higher NLR have a considerably inferior OS and PFS than the control group.

Studies indicated by an asterisk (*) represent meta-analyses. ATC, Anaplastic Thyroid Cancer; BC, Breast Cancer; CAR, C-Reactive Protein-to-Albumin Ratio; CRC, Colorectal Cancer; CSS, Cancer-Specific Survival; DFS, Disease-Free Survival; DTC, Differentiated Thyroid Carcinoma; F-NLR, Fibrinogen-Neutrophil-to-Lymphocyte Ratio; HCC, Hepatocellular Carcinoma; HER2, Human Epidermal Growth Factor Receptor 2;mCRPC, Metastatic Castration-Resistant Prostate Cancer; NACT, Neoadjuvant Chemotherapy; NSCLC, Non-Small Cell Lunger Carcinoma; NLR, Neutrophil-to-Lymphocyte Ratio; NLRc, Postoperative NLR/Preoperative NLR; OS, Overall Survival; PAC, Pancreatic Adenocarcinoma; pCR, Pathological Complete Response; PDTC, Poorly Differentiated Thyroid Cancer; PFS, Progression-Free Survival; PLR, Platelet-to-Lymphocyte Ratio; RFS, Relapse-Free Survival; RDW, Red Cell Distribution Width; R-NLR, Red Blood Cell Distribution Width-NLR; RT, Radiotherapy; SCLC, Small Cell Lung Carcinoma.

The findings of these studies highlight the importance of considering NLR in the clinical management of cancer patients. Early diagnosis and detection of cancer is critical for providing effective treatment strategies and improving patient outcomes. Thus, the identification and utilization of prognostic indicators like NLR can play a crucial role in the decision-making process of healthcare providers. Furthermore, the growing body of evidence on the prognostic value of NLR highlights the need for further research to fully understand its mechanisms and applications in cancer care.

In light of these findings, it is suggested that healthcare providers should include NLR as a part of their routine assessments for cancer patients. Additionally, further longitudinal research is needed to determine the optimal cutoff values for NLR and to assess its utility in different cancer populations. This will not only aid in the development of more effective treatment strategies but also improve patient outcomes. Overall, the results of the studies discussed in this review suggest that the prognostic potential of NLR in cancer is a promising area for further investigation and clinical application.

## Author contributions

KH-G: Conception of the work, conducting the study, reviewing the literature, drafting the work and revising it. VS: Conducting the study, reviewing the literature, drafting the work and revising it. AH: Conducting the study, drafting the work and revising it, reviewing the literature. AM: Conducting the study, drafting the work and revising it. SN: Conception of the work, conducting the study, drafting the work and revising it. SR: Conception of the work, conducting the study, reviewing the literature, drafting the work and revising it. All authors contributed to the article and approved the submitted version.

## Glossary

**Table:**

ACT	Anaplastic Thyroid Cancer
BC	Breast Cancer
BCLC	Barcelona Clinic Liver Cancer
CBC	Complete Blood Count
CAR	C-Reactive Protein-to-Albumin Ratio
CRC	Colorectal Cancer
CSS	Cancer-Specific Survival
DFS	Disease-Free Survival
DTC	Differentiated Thyroid Cancer
ESD	Endoscopic Submucosal Dissection
F-NLR	Fibrinogen-Neutrophil-to-Lymphocyte Ratio
HCC	Hepatocellular Carcinoma
HER2	Human Epidermal Growth Factor Receptor 2
HGSC	High-Grade Serous Ovarian Cancer
LC	Lung Cancer
MetS	Metabolic Syndrome
mCRPC	Metastatic Castration-Resistant Prostate Cancer
NACT	Neoadjuvant Chemotherapy
NET	Neutrophil Extracellular Trap
NLR	Neutrophil-to-Lymphocyte Ratio
NLRc	Postoperative NLR/Preoperative NLR
NSCLC	Non-Small Cell Lung Cancer
OS	Overall Survival
PAC	Pancreatic Adenocarcinoma
PDTC	Poorly Differentiated Thyroid Cancer
pCR	Pathological Complete Response
PFS	Progression-Free Survival
PLR	Platelet-to-Lymphocyte Ratio
PSA	Prostate-Specific Antigen
R-NLR	Red Blood Cell Distribution Width - Neutrophil-to-Lymphocyte Ratio
RDW	Red Cell Distribution Width
RCC	Renal Cell Carcinoma
RFS	Relapse-Free Survival
RT	Radiotherapy
SCLC	Small Cell Lung Cancer
TAN	Tumor-Associated Neutrophil
